# Pain-QuILT: Clinical Feasibility of a Web-Based Visual Pain Assessment Tool in Adults With Chronic Pain

**DOI:** 10.2196/jmir.3292

**Published:** 2014-05-12

**Authors:** Chitra Lalloo, Dinesh Kumbhare, Jennifer N Stinson, James L Henry

**Affiliations:** ^1^Medical Sciences Graduate ProgramFaculty of Health SciencesMcMaster UniversityHamilton, ONCanada; ^2^Toronto Rehabilitation InstituteDivision of Physical Medicine and RehabilitationUniversity of TorontoToronto, ONCanada; ^3^Department of Child Health Evaluative Sciences, The Hospital for Sick ChildrenLawrence S Bloomberg, Faculty of Nursing, University of TorontoToronto, ONCanada; ^4^Faculty of Health SciencesMcMaster UniversityHamilton, ONCanada

**Keywords:** chronic pain, assessment tool, Internet, clinical feasibility

## Abstract

**Background:**

Chronic pain is a prevalent and debilitating problem. Accurate and timely pain assessment is critical to pain management. In particular, pain needs to be consistently tracked over time in order to gauge the effectiveness of different treatments. In current clinical practice, paper-based questionnaires are the norm for pain assessment. However, these methods are not conducive to capturing or tracking the complex sensations of chronic pain. Pain-QuILT (previously called the Iconic Pain Assessment Tool) is a Web-based tool for the visual self-report and tracking of pain (quality, intensity, location, tracker) in the form of time-stamped records. It has been iteratively developed and evaluated in adolescents and adults with chronic pain, including usability testing and content validation. Clinical feasibility is an important stepping-stone toward widespread implementation of a new tool. Our group has demonstrated Pain-QuILT clinical feasibility in the context of a pediatric chronic pain clinic. We sought to extend these findings by evaluating Pain-QuILT clinical feasibility from the perspective of adults with chronic pain, in comparison with standard paper-based methods (McGill Pain Questionnaire [MPQ] and Brief Pain Inventory [BPI]).

**Objective:**

The goal of our study was to assess Pain-QuILT for (1) ease of use, (2) time for completion, (3) patient preferences, and (4) to explore the patterns of self-reported pain across the Pain-QuILT, MPQ, and BPI.

**Methods:**

Participants were recruited during a scheduled follow-up visit at a hospital-affiliated pain management and physical rehabilitation clinic in southwestern Ontario. Participants self-reported their current pain using the Pain-QuILT, MPQ, and BPI (randomized order). A semistructured interview format was used to capture participant preferences for pain self-report.

**Results:**

The sample consisted of 50 adults (54% female, 27/50) with a mean age of 50 years. Pain-QuILT was rated as significantly easier to use than both the MPQ and BPI (*P*<.01) and was also associated with the fewest difficulties in completion. On average, the time to complete each tool was less than 5 minutes. A majority of participants (58%, 29/50) preferred Pain-QuILT for reporting their pain over alternate methods (16%, 8/50 for MPQ; 14%, 7/50 for BPI; 12%, 6/50 for “other”). The most commonly chosen pain descriptors on MPQ were matched with Pain-QuILT across 91% of categories. There was a moderate-to-high correlation between Pain-QuILT and BPI scores for pain intensity (*r*=.70, *P*<.01).

**Conclusions:**

The results of this clinical feasibility study in adults with chronic pain are consistent with our previously published pediatric findings. Specifically, data indicate that Pain-QuILT is (1) easy to use, (2) quick to complete, (3) preferred by a majority of patients, and (4) correlated as expected with validated pain measures. As a digital, patient-friendly method of assessing and tracking pain, we conclude that Pain-QuILT has potential to add significant value as one standard component of chronic pain management.

## Introduction

Chronic pain, defined as pain that persists beyond normal time of healing, is a prevalent and debilitating problem that is now recognized as a disease [1-3]. Common types of chronic pain include low back, headache, abdominal, musculoskeletal, and neuropathic pain [4]. Pain is a complex sensory and emotional phenomenon that, while intensely experienced, is often difficult to communicate [5].

Accurate and timely pain assessment is critical to developing and monitoring a pain management plan [6]. Given that there is no medical test to directly measure pain, health care providers rely primarily on patient self-report, including pain quality (what it feels like), intensity (how much it hurts), location (spatial distribution), and temporal nature (how it changes over time) [6]. Assessment of pain quality and location is particularly important because this information can be used to distinguish between different diagnostic subgroups (eg, neuropathic versus non-neuropathic pain) [7,8].

Chronic pain management often takes place across multiple settings (eg, hospitals, clinics) and involves numerous health care providers, including physicians, nurses, physiotherapists, chiropractors, and psychologists [9-11]. Pain outcomes need to be consistently tracked over time in order to gauge the effectiveness of different management strategies, including physical, psychological, and pharmacological approaches. However, there is often a lack of consistency in the assessment of pain across these different settings and providers. One reason for this lack of consistency is the standard use of paper-based assessment tools, which are not conducive to tracking pain over time. Commonly used paper-based tools include the McGill Pain Questionnaire (MPQ) [12] and the Brief Pain Inventory (BPI) [13]. However, there is limited research on the serial use of these measures in clinical pain assessment.

The emergence of Internet and mobile technology has created opportunities for innovation in the field of pain assessment and management. For example, electronic pain diaries offer advantages such as ease of data tracking, improved patient compliance, and capture of real-time pain reports without memory bias [14-20].

Pain-QuILT is a Web-based tool for the visual self-report and tracking of pain (quality, intensity, location, tracker) in the form of time-stamped records [21-24]. Pain quality is expressed by choosing from a validated library of labeled pain icons, such as a matchstick for “burning pain”. Pain intensity is quantified using a 0-10 numerical rating scale (NRS) ranging from “no pain” to “worst pain imaginable”. Pain location is illustrated by “dragging-and-dropping” pain icons onto a detailed virtual body-map that is codified into over 100 regions.

To our knowledge, Pain-QuILT is the only tool that captures the complex sensations of chronic pain by allowing patients to self-report different qualities and intensities of pain across their entire body. For example, they can record the simultaneous experience of a “3/10” burning pain in their shoulder as well as a “5/10” pain in their foot that is both “burning” and “sharp”. All reported data are digitally captured and then populated into a database, which can be used to track changes in pain quality and intensity across different body regions over time.

Health care professionals can use this information to monitor the effectiveness of any pain management practices. Patients can keep track of their pain to help inform self-management in the home setting. By standardizing the assessment of pain outcomes in a digitized format, Pain-QuILT may also improve the coordination of pain management across multiple health care providers.

Pain-QuILT has been iteratively developed and evaluated in adolescents and adults with chronic pain, including usability testing and content validation. Before widespread implementation of Pain-QuILT, it is critical to evaluate clinical feasibility (ie, the ease with which it can be applied in a real-world setting), compared with standard methods of pain assessment. Recently, our group established clinical feasibility in an interdisciplinary pediatric chronic pain clinic that used a semistructured interview method to assess pain [24]. In comparison with this standard method, Pain-QuILT was preferred by a majority of adolescent patients and was perceived to be clinically useful for visually capturing pain and promoting better communication between patients and health providers.

Given that the MPQ and BPI are the standard tools used in adults, the purpose of this study was to extend the findings from our pediatric work to evaluate clinical feasibility of Pain-QuILT among adults with chronic pain in comparison with the MPQ and BPI. In the context of this clinical feasibility study, our primary aims were to assess Pain-QuILT for (1) ease of use, (2) time for completion, and (3) patient preferences. Our secondary aim was to explore the patterns of self-reported pain across the comparator methods of Pain-QuILT, MPQ, and BPI.

## Methods

### Study Setting

This study was conducted at a hospital-affiliated pain management and physical medicine and rehabilitation outpatient clinic in southwestern Ontario. It was staffed by an interdisciplinary team of health care professionals, consisting of a physiatrist, physical therapist, and kinesiologist. Patients who are referred to this outpatient clinic receive a thorough medical evaluation, including assessment of pain, and are then informed of the management plan including pharmacological, injection, and physical therapies. They may also be referred for psychological therapy (eg, group counseling, cognitive behavioral therapy) if needed. All patients are reassessed at timely intervals and treatments are adjusted according to clinical need.

### Recruitment

Informed written consent was obtained from all participants, and the study was approved by the locally responsible Research Ethics Boards. A health care provider known to patients identified eligible individuals by screening the patient lists of consecutively scheduled clinic appointments. Individuals were eligible to participate if they were (1) aged 18 years or older, (2) able to speak and read English, and (3) currently experiencing pain of any intensity according to self-report. Individuals were excluded if they had severe cognitive impairment or major comorbid medical or psychiatric illness that could preclude their ability to self-report pain or take part in a verbal interview, according to their health care provider. Individuals were also excluded if they had severe vision or hand dexterity impairments that could prevent independent use of a computer and mouse.

### Demographic and Health-Related Data

Following consent, each participant completed a Demographic and Health Questionnaire, which collected data on age, sex, computer comfort, weekly computer use, language proficiencies, education level, and date of pain problem onset.

### Interview Protocol

All participants took part in an individual semistructured interview (20-30 minutes) with a trained investigator (author CL). The investigator was experienced in conducting qualitative interviews and used techniques to minimize the power differential between the interviewer and participant (eg, established rapport, engaged in active listening, used relaxed body language) [25]. The investigator also stressed that the research team wished to ensure that Pain-QuILT addressed the needs of adults with chronic pain and thus encouraged participants to freely express opinions about good and bad aspects of the tool. As a first step, participants self-reported their pain using the Pain-QuILT, MPQ, and BPI (described in detail below). These tools were administered in a randomized order for each participant, in order to minimize potential order effects. Investigator observation and participant comments were used to identify any difficulties or confusion with using each tool; these were recorded as field notes. The time required to complete each tool was recorded. Next, a semistructured interview format was used to discuss participant preferences for pain self-report. A 0-10 NRS ranging from “not easy at all” to “very easy” was used to appraise each tool. Qualitative written feedback on the ease of using each tool was also collected. Finally, participants were asked to indicate their preference of methods for self-reporting pain and explain the reason for their choice. All interviews were conducted by the same investigator in a quiet room within the clinic.

### Pain Tool Comparison

#### McGill Pain Questionnaire

This paper-based questionnaire was developed in the 1970s through groundbreaking research that was focused on identifying common word descriptors for the pain experience [12,26,27]. At the time, there was no available tool that accounted for the multidimensional nature of pain. The MPQ is composed of 20 subclasses that correspond to sensory, affective, evaluative, and miscellaneous pain. Each subclass consists of a clustered list of 2-6 word descriptors. For example, the first subclass of word descriptors is “sensory-temporal” and is made up of the descriptors: “flickering; quivering; pulsing; throbbing; beating; pounding”. There is a total of 78 descriptors on the MPQ. Participants were instructed to review each discrete cluster of words and then select the one word that best described their current pain. If none of the words within a cluster were descriptive of their pain, then no word was selected. The MPQ is one page in length and was administered by the study investigator.

#### Brief Pain Inventory Short Form

This paper-based questionnaire was developed in the 1980s for patients with cancer pain, based on research suggesting that existing measures such as the MPQ were burdensome for patients to complete [13,28]. Since its initial development, the BPI has subsequently become one of the most widely used tools for assessing all types of pain in both clinical and research settings [29]. It is designed to assess pain location and severity as well as level of interference with daily life. In the present study, participants used a pen to shade painful areas on a body-manikin diagram. The body-manikin consisted of anterior and posterior aspects and included no regional demarcations. Next, participants were required to rate the intensity of their “pain right now” as well as their “worst”, “least”, and “average” pain from the past 24 hours using separate 0-10 NRS items ranging from “no pain” to “pain as bad as you can imagine”. Finally, participants were asked to rate the extent to which pain had interfered with different parts of their life in the past 24 hours. Each quality of life domain was rated on a separate NRS ranging from 0 (“does not interfere”) to 10 (“completely interferes”). The BPI is one page in length and was administered by the study investigator.

#### Pain-QuILT

**Figure 1:**
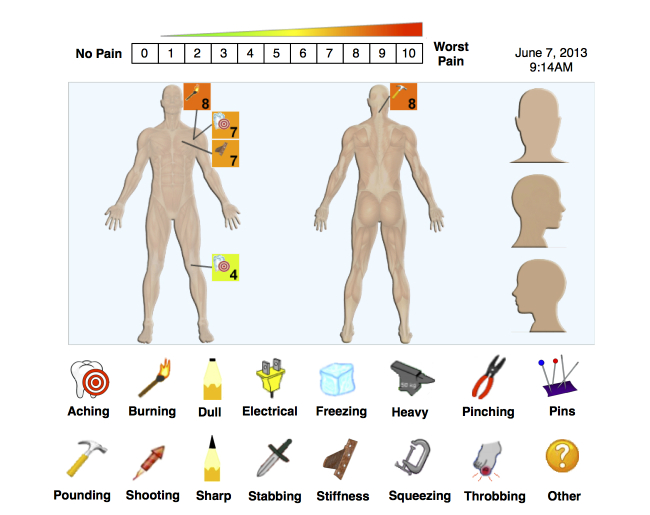
Screenshot of Pain-QuILT user interface for self-reporting the quality, intensity, and location of current pain. Copyright McMaster University. Used with permission. All permission requests for this image should be made to the copyright holder (McMaster Industry Liaison Office).

Participants were taught how to use Pain-QuILT via a standard 3-minute demonstration. Following confirmation of understanding, each participant was instructed to use the investigator laptop computer (MacBook Pro) with external mouse to “create a picture” of their current pain, as illustrated in Figure 1. First, they chose from the library of labeled pain quality icons to describe what their pain felt like. The Pain-QuILT library consisted of 16 icons to represent aching, burning, dull, electrical, freezing, heavy, pinching, pins & needles, pounding, shooting, sharp, stabbing, stiffness, squeezing, throbbing, and “other” pain. They then used the mouse to “drag-and-drop” a miniature copy of this descriptive icon onto a virtual body-map to show pain location. The entire body-map was displayed on a single screen and was made up of anterior and posterior aspects, as well as magnified views of the head (anterior, posterior, side-view). The body-map was codified into 110 distinct regions, and each region became highlighted in blue as the computer mouse hovered over it. Next, after “dropping” the icon onto the appropriate body region, the user assigned a rating of intensity for this pain by using a “pop-up” 0-10 NRS ranging from “no pain” to “worst pain imaginable”. The 0-10 NRS also corresponded with a color scale ranging from green (lower intensity) to red (higher intensity). After the user had chosen an intensity value, the pain icon was added to the body-map, along with the numerical rating. The dropped icon-number pair was enclosed within a square box whose fill color corresponded to the intensity rating (eg, dark green fill for a rating of 1/10). Users continued to “drag-and-drop” numbered icons onto the virtual body-map until all of their current pain or pains had been recorded. Figure 1 shows a patient reporting multiple pains across their body of different qualities and intensities, specifically, shoulder pain that is both “burning” and “aching”, a painful stiffness in their chest, an “aching” knee pain, and a “pounding” pain in the back of their neck. All user-entered pain data (quality, intensity, location), as well as information on time and date of entry, were automatically uploaded to a back-end database that was accessible to the research team.

### Data Analysis

Qualitative written data and field notes from the semistructured interview were transcribed verbatim and imported into the qualitative software program, HyperRESEARCH [30]. This software was used to facilitate a simple content analysis of the data [31]. A line-by-line coding analysis was used to identify key concepts from the interview transcripts and field notes. Concepts addressed during the semistructured interviews were used to thematically code and organize participant responses [31]. Participant quotations were selected to illustrate each key interview concept with the aim of representing the balance of opinion among participants.

Quantitative data from the Demographic and Health Questionnaire, MPQ, BPI, and Pain-QuILT were coded, scored, and entered into a Statistical Package for the Social Sciences database [32]. As described by Lalloo and colleagues, the extracted parameters from each Pain-QuILT report were the number of unique painful sites (range 0 to 110) and number of different pain quality descriptors (range 0 to 16) used to express current pain [24]. Additionally, a cumulative mean pain intensity score was calculated across all painful body sites. While this cumulative score provided a convenient indicator of the central tendency of data, it was also sensitive to outliers. Thus, we also extracted the lowest and highest single NRS intensity score to provide an indicator of data dispersion. For example, if a participant reported a 5/10 burning pain in their foot, a 3/10 burning pain in their hand, and a 3/10 stabbing pain in their back, then the number of unique painful sites would be recorded as 3, the number of unique pain quality descriptors would be 2, the cumulative intensity score would be calculated as [(5+3+3)/3]=3.7, the lowest reported NRS score would be 3, and the highest reported NRS score would be 5.

All data were analyzed descriptively to assess measures of central tendency (mean, median) and dispersion [standard deviation, interquartile range]. Data were also evaluated to ensure that they met the assumptions of parametric statistical analysis (ie, the normal distribution). When these assumptions were not met, the non-parametric equivalent test was used. Repeated measure analysis of variance (ANOVA) was used to determine whether there were any differences between Pain-QuILT, MPQ, and BPI in terms of time to complete or ease of use ratings. Pearson correlations were used to examine the association between pain intensity scores on Pain-QuILT and BPI*.* The *a priori* criterion for evidence of convergent validity was a moderate correlation of *r*=.5 between Pain-QuILT and BPI scores for current pain intensity. Using the guidelines from Streiner and Norman pertaining to sample size for correlation coefficients, assuming alpha=.05 and beta=.05, the required sample size was N=50 [33]. The level of significance was set at *P*<.05 for all tests.

##  Results

### Participant Characteristics

A total of 50 adults completed the study over a 5-month period in 2013. Sample characteristics are summarized in Table 1. Nearly all participants (48/50, 96%) had a computer at home as well as Internet access (45/50, 90%). Of the 50 participants, 84% (42/50) reported being “comfortable” or “very comfortable” with using computers, while 10% (5/50) were “a little comfortable” and 6% (3/50) were “not at all comfortable”. The self-reported frequency of computer use among participants was none (3/50, 6%), once per week (3/50, 6%), twice per week (2/50, 4%), three times per week (4/50, 8%), five times per week (1/50, 2%), and every day (37/50, 74%).

**Table 1:** Characteristics of study participants (N=50).

Characteristics		n
Age in years, mean (SD) (range)		50 (14) (18-76)
**Gender, n (%)**		
	Male	23 (46)
	Female	27 (54)
**Language, n (%)**		
	English as first spoken language	39 (78)
	Spoke English only	31 (62)
	Spoke English and another language	19 (38)
Total years education, mean (SD) (range)		13.8 (3.8) (0-21)
Chronic pain duration in years, mean (SD) (range)		8.3 (8.9) (1-33)
**Current pain treatment modalities, n (%)**		
	Pharmacological	43 (86)
	Physical therapy	19 (38)
	Massage therapy	9 (18)
	Alternative or complementary	5 (10)
	Chiropractic therapy	2 (4)
	Acupuncture	2 (4)
**Pain interference in past 24 hours, mean (SD )**		
	Normal work	7.2 (2.5)
	Enjoyment of life	7.0 (2.9)
	Sleep	6.8 (2.6)
	General activity	6.7 (2.5)
	Mood	6.2 (2.9)
	Walking ability	5.6 (3.1)
	Relations with other people	5.0 (2.9)

### Self-Reported Pain

#### McGill Pain Questionnaire

**Figure 2:**
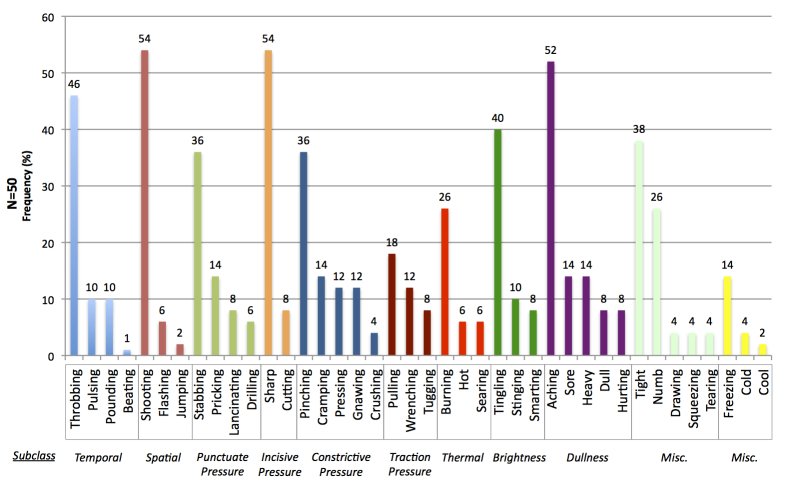
Relative frequency of words chosen by participants on the McGill Pain Questionnaire to describe their current pain.

The relative endorsement of MPQ pain quality descriptors between and within subclasses is illustrated in Figure 2. The most commonly chosen MPQ words to express current pain were matched with a descriptor in the Pain-QuILT library across all subclasses, except for “miscellaneous”. This pattern was consistent regardless of whether the MPQ was administered before Pain-QuILT (29/50, 58%), or Pain-QuILT was administered before the MPQ (21/50, 42%).

#### Brief Pain Inventory

The mean score for current reported pain intensity was 6.6 (SD 2.1). The mean scores for recalled pain in the past 24 hours were 7.9 (SD 1.4) for “worst” pain, and 4.4 (SD 2.2) for “least” pain, respectively.

#### Pain-QuILT

**Figure 3:**
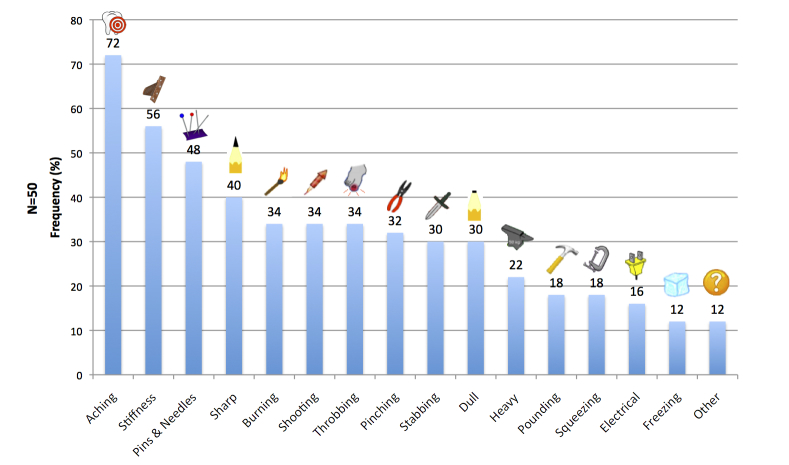
Relative frequency of Pain-QuILT icons chosen by participants to describe their current pain.

The mean number of unique painful sites reported was 6.5 (SD 4.0, range 1-22). The mean number of different pain qualities used to describe current pain was 5.0 (SD 2.4, range 1-10). The relative endorsement of *Pain-QuILT* icons across all participants is illustrated in Figure 3. The mean reported intensity for current pain (ie, the cumulative calculated score across all body sites) was 6.2 (SD 2.0). The mean lowest reported pain intensity score was 4.8 (SD 2.1), and the mean highest reported pain intensity score was 7.4 (SD 2.1).

The Pearson correlation coefficient between the *Pain-QuILT* score for current pain (calculated across all body sites) and BPI score for current pain (single NRS rating) was *r*=.70. The Pearson correlation coefficient between the highest reported intensity score on Pain-QuILT and the BPI score for current pain was *r*=.76. The Pearson correlation coefficient between the lowest reported intensity score on Pain-QuILT and the BPI score for current pain was *r*=.55.

### Ease of Use

All participants reported the relative ease of using each tool for self-reporting pain. The mean ratings were 5.9 (SD 2.6) for the MPQ, 7.0 (SD 2.6) for the BPI, and 8.3 (SD 2.0) for Pain-QuILT. Overall, there was a significant difference between the tools in terms of perceived ease of use, *F*_2,96_=20.6, *P*<.001. Pairwise comparisons also indicated significant differences between the MPQ and BPI (*P*=.009), MPQ and Pain-QuILT (*P*<.001), as well as BPI and Pain-QuILT (*P=*.002).

### Participant-Reported Difficulties With Using Each Pain Tool

Overall, 46% (23/50) of participants indicated that they had difficulties in completing the MPQ, while 22% (11/50) reported difficulties with the BPI and 16% (8/50) specified difficulties with Pain-QuILT.

The most commonly reported issue with the MPQ was trouble with understanding the qualitative word descriptors (10/23, 43%) due to language barriers (eg, English as second language) or uncommon vocabulary, such as “taut” and “smarting”. Participants (7/23, 30%) also reported that the available pain words “...weren’t very good to describe [pain]” (ie, lack of descriptiveness). Other participants (7/23, 30%) noted that it was difficult to select the right words to express their pain due to ambiguity (“what is difference between cool, cold, freezing?”), the number of available options (“too many choices”), and the presence of more than one relevant word from certain subclasses. Last, participants (2/23, 9%) expressed concern about potentially misrepresenting their pain to their health care providers: “more fear of not describing your pain properly with this test”.

The most commonly reported issues with the BPI were communicating pain location using the body-manikin (2/11, 18%; “hard to pull out meaning”) and choosing intensity ratings to describe pain (2/11, 18%; “hard time with pain numbers”). Other reported difficulties included recalling pain over the last 24 hours (“hard to simplify pain”), reporting pain from multiple sites (“varying intensities of pain from different injuries”), and questionnaire design (“cumbersome to complete, too general”). One participant also indicated a “fear of not explaining properly what is happening”.

The most commonly reported issues with Pain-QuILT were related to the virtual body-map (3/8, 37.5%). Specifically, participants identified a need for orientation labels (left, right) and to make it easier to isolate specific painful body areas (“hard to find specific regions on [the] back versus a ‘paint’ tool, because some pain radiates”). In addition, participants (2/8, 25%) indicated difficulty in choosing pain quality icons due to “too many choices...sometimes it aches, sometimes it burns”, and a dislike of using descriptors because “pain just hurts”. Other participants (3/8, 37.5%) identified a “bug” in the software related to an inability to remove icons that were mistakenly added to the body-map.

### Time to Complete

The mean time required by participants to complete a single pain report using each tool was 4.2 minutes (SD 1.5) for the MPQ, 4.0 minutes (SD 1.4) for the BPI, and 4.1 minutes (SD 2.2) for Pain-QuILT. There was no significant difference between the tools in terms of time to complete, *F*_1.4,44.8_=0.13, *P*=.81.

### Participant Preferences for Self-Reporting Pain

Overall, 16% (8/50) participants chose the MPQ as their preferred method for self-reporting pain, while 14% (7/50) chose the BPI, and 58% (29/50) chose Pain-QuILT. Four of the 50 participants (8%) indicated that they preferred the “other” method of verbally explaining pain to their health care provider. Finally, one participant (1/50, 2%) indicated an equal preference between the BPI and Pain-QuILT.

Reasons for selecting the MPQ included a preference for paper versus electronic pain reporting and greater perceived precision in describing pain, for example, “[it has] words that exactly indicate what is happening to [my] leg—bang on”.

Reasons for choosing the BPI included familiarity, the ability to describe how pain changes over time, and ease of choosing ratings on a set scale, for example, “[it] seems more easy to answer personally. Fits the way that I speak”.

Explanations for choosing Pain-QuILT included greater ease of use, ability to pinpoint different locations and types of pain, preference for computer versus paper-based pain reporting, as well as the visual language to express pain, for example, “[I would] feel more confident being treated by a doctor if they used this tool because [they] would know exactly what you are feeling”.

## Discussion

### Previous Findings

Our previous work has established the acceptability, usability, and content validity of Pain-QuILT in samples of adults with central post-stroke pain [21], adults in the community with a range of different types of chronic pain [22], as well as adults and adolescents with arthritis pain [34]. Clinical feasibility testing, the focus of the present study, is an important stepping-stone toward widespread implementation of a new assessment tool [35]. Our group has recently demonstrated clinical feasibility of Pain-QuILT in the context of an interdisciplinary pediatric chronic pain clinic among adolescents aged 12-18 years [24]. The present study sought to extend these findings by evaluating clinical feasibility of Pain-QuILT from the perspective of adults attending an outpatient pain clinic for treatment of chronic pain. This study included a comparison of Pain-QuILT with standard methods of pain assessment.

### Principal Results

As a tool for self-reporting pain, Pain-QuILT was rated as significantly easier to use than the MPQ and BPI, which are two of the most commonly used pain assessment tools in research and clinical practice. Almost half (46%) of participants reported difficulties in using the MPQ. Most of these difficulties related to understanding the pain descriptors and finding accurate words to express pain from a large number of options. These findings of the present study are consistent with a meta-analysis of 51 studies involving 3624 patients, which found that most MPQ words (75%) are rarely endorsed by patients to describe their pain [36]. Although the BPI was associated with fewer reported difficulties, participants indicated that its design was not conducive to reporting different intensities of pain in different body sites*.* Numerous studies have demonstrated that chronic pain is rarely confined to a single body region [37-39]. For instance, in a study involving 2445 patients, Carnes and colleagues found that 73% experienced pain across multiple body sites [37]. Among patients with low back pain, only 13% experienced regionally isolated pain. In terms of implications for pain assessment and management, these authors concluded, “self-reported measures of multi-site pain are problematic with pain measures that are site-specific. Pain in other areas may render them less reliable and responsive. Future intervention studies should consider recording other pain sites to identify predictors of response to treatment” (p. 1170) [37]*.* Overall, Pain-QuILT was associated with the fewest reported difficulties among participants. Most of the identified issues (75%) will be resolved in the next iteration of Pain-QuILT software (eg, adding orientation labels to body map, fixing “bug” related to deleting unwanted icons). Participant concerns related to the changing nature of pain (“sometimes it aches, sometimes it burns”) will be addressed in future longitudinal studies, which will allow patients to use Pain-QuILT as a diary to document symptoms as they occur. A major identified strength of Pain-QuILT was the ability to record multiple sites, types, and intensities of pain.

The average time required to complete each assessment tool was less than 5 minutes. While there was no significant time difference between the tools, it is important to note that patients can enter Pain-QuILT data independently, while the MPQ and BPI are usually administered by a health care provider in the context of a clinic appointment. Moreover, Pain-QuILT data are generated and stored in a digital format, while information from MPQ and BPI must be manually transcribed into a spreadsheet (paper or computer-based) in order to facilitate tracking over time. Thus, Pain-QuILT has the potential to increase efficiency of clinic appointments by (1) empowering patients to self-report pain on their own time (eg, at home and/or in the clinic waiting room), (2) providing health care providers with digital summaries of tracked pain data to evaluate and inform their management plan, and (3) standardizing the assessment of pain outcomes for use across multiple providers.

Given the inherently personal nature of pain, it is important to consider patient preferences regarding the most effective way of expressing symptoms. The majority of participants (58%) indicated positive preference for Pain-QuILT over alternate methods. It is well recognized that patient engagement is a critical factor in the successful management of chronic disease [40]. In particular, effective doctor-patient communication is known to enhance the health outcomes of pain management [41]. The interactive and dynamic format of Pain-QuILT may also help patients forge a stronger emotional connection to the tool as a means for portraying and conveying their pain experience, compared to static questionnaires. Moreover, there is a growing body of literature documenting the rise of “self-tracking” among people living with chronic illness. A recent Pew Research Center report found that 40% of adults with 1 chronic condition and 62% of adults with 2 chronic conditions currently self-track their symptoms [42]. In terms of patient benefits, respondents indicated that self-tracking influenced their overall approach to maintaining health (56%), prompted them to ask their doctor new questions (53%), or influenced a treatment decision (45%) [42]. Thus, by providing a user-friendly method for communicating with health care providers and self-tracking painful symptoms, Pain-QuILT may encourage greater patient involvement in the long-term management of their own disease.

There is a growing number of patient-oriented mobile applications (apps) designed to aid the self-tracking of pain. In 2011, Rosser and Eccleston identified 111 pain management apps, of which 24% included a self-monitoring function [43]. A more recent scoping review, conducted in 2013, identified 224 pain apps, of which 14% allowed users to self-track their symptoms [44]. Unfortunately, both studies identified major limitations in the current field of pain apps, including a lack of formal evaluation and limited involvement of health care professionals and patients in their development. Pain-QuILT has been iteratively evaluated and refined through consultation with patients as well as health care professionals and thus has potential to address these identified gaps in the field, as one component of chronic pain management.

Given that participants were asked to self-report their current pain using three different methods, we expected to observe consistency in reported pain. Using the MPQ, participants were presented with a choice of 46 qualitative descriptors across 11 subclasses. Interestingly, the most frequently chosen MPQ words were consistent with the icon descriptors on Pain-QuILT. This relationship was independent of the order of tool assessment. Pain-QuILT icons and word descriptors have been iteratively refined based on patient interviews to ensure that they are representative of the pain experience. The observation that the icons correspond with the most frequently endorsed MPQ descriptors provides further evidence of validity. In terms of pain intensity scores, we examined correlations between Pain-QuILT (body site-specific pain scores) and the BPI (single global score for current pain). There were high correlations (*r*≥.70) observed between BPI score and (1) the calculated average pain score across all body sites, and (2) the single highest reported pain score across all body sites. There was also a moderate correlation (*r*=.55) observed between BPI score and the single lowest reported pain score across all body sites. Along with our previous pediatric study, which compared Pain-QuILT scores with a verbal NRS (*r*=.61), the current data provides further evidence of convergent validity. Importantly, in terms of clinical usefulness, we suggest that the greater level of detail elicited by Pain-QuILT may help inform pain management strategies (eg, observing how treatment affects pain quality and intensity scores within specific body sites) more than a single global intensity score.

### Limitations and Future Directions

The present clinical feasibility study was conducted at a single interdisciplinary pain management and rehabilitation clinic in Southwestern Ontario. Although the organization and treatment model of this site was consistent with other Canadian multidisciplinary pain treatment facilities [45], we acknowledge that future work is needed to evaluate clinical feasibility of Pain-QuILT in other settings. Further, given the interview component of this study, it was necessary for all participants to be able to speak and read English. Although 38% of participants spoke multiple languages, future work is needed to formally evaluate Pain-QuILT in non-English speaking groups. Given the visual nature of Pain-QuILT reporting, it could prove to enhance pain communication for individuals with limited verbal or cognitive skills.

Participants in this study completed only a single Pain-QuILT report. Future work will evaluate whether patient perceptions regarding ease of use and preferences, as well as time to complete, are affected by repeated usage.

### Conclusions

The results of this clinical feasibility study in adults with chronic pain are consistent with our previously published pediatric findings [24]. Specifically, data indicate that Pain-QuILT is (1) easy to use, (2) quick to complete, (3) preferred by a majority of adults with chronic pain, and (4) correlated as expected with validated pain measures. As a digital, patient-friendly method of assessing and tracking pain, we conclude that Pain-QuILT has potential to add significant value as one standard component of chronic pain management.

The tool will be licensed for clinical use and research studies through the McMaster Industry Liaison Office [46,47]. Updated information on availability will be provided on the author website [47] and Twitter account (@PainQuILT).

## Abbreviations

BPI: Brief Pain Inventory
MPQ: McGill Pain Questionnaire
NRS: Numerical Rating Scale
SD: standard deviation
